# Supplementary material for: Characterization of P-Glycoprotein Inhibitors for Evaluating the Effect of P-Glycoprotein on the Intestinal Absorption of Drugs
Source: Pharmaceutics. 2021 Mar 15;13(3):388. doi: 10.3390/pharmaceutics13030388 (PMC7999658; doi:10.3390/pharmaceutics13030388)
Supplement: Supplementary file 1 [file pharmaceutics-13-00388-s001.pdf]

pharmaceutics

Supplementary Materials: Characterization of P-glycoprotein Inhibitors for Evaluating the Effect of P-glycoprotein on the Intestinal Absorption of Drugs

Yusuke Kono [1], Iichiro Kawahara [2,†], Kohei Shinozaki [2], Ikuo Nomura [2], Honoka Marutani [1], Akira Yamamoto [2] and Takuya Fujita [1,*]

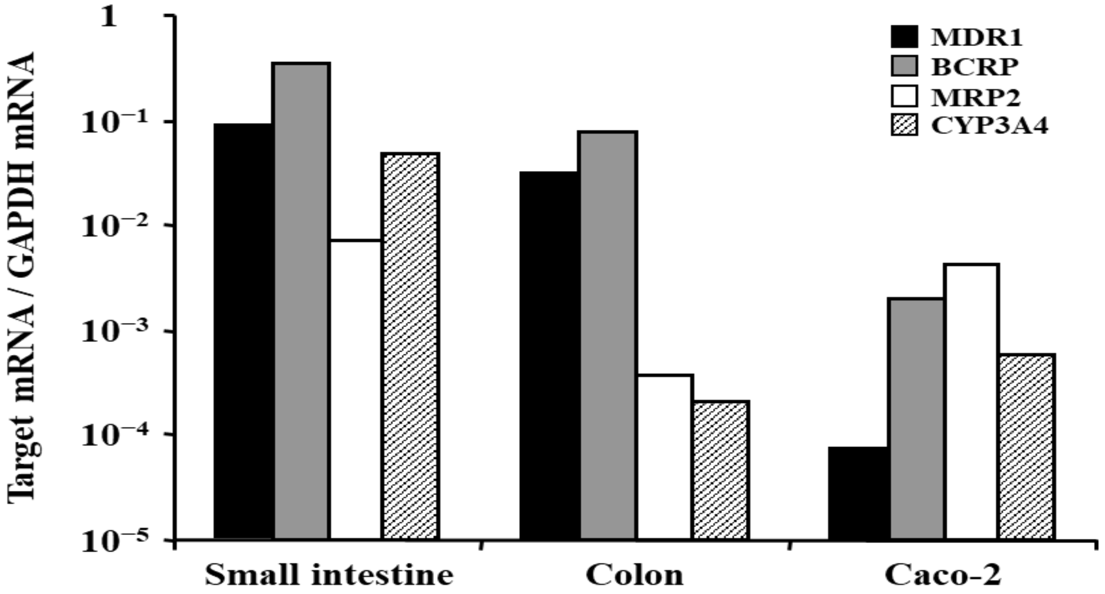

Figure S1. Relative mRNA expression levels of MDR1, BCRP, MRP2, and CYP3A4 in human small intestine, colon and Caco-2 cells. Relative mRNA expression levels were determined by real-time RT-PCR. GAPDH was selected as an endogenous RNA control to normalize for difference in amount of total RNA.

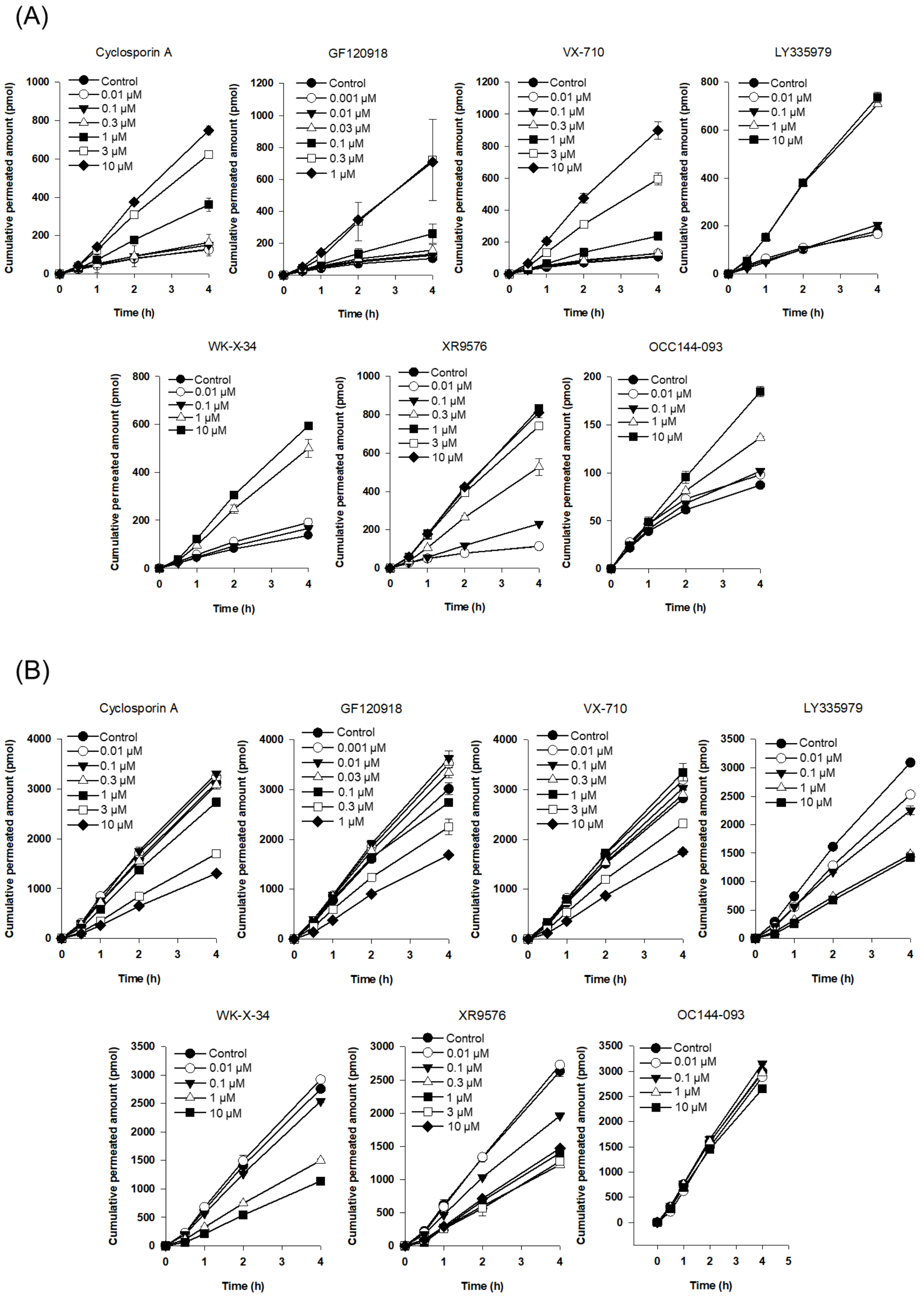

Figure S2. Bidirectional transport of paclitaxel across Caco-2 cell monolayers**.** The apical-to-basal (A) and basal-to-apical (B) transport of paclitaxel (5 μM) in the presence or absence of various concentrations of P-gp inhibitors. Data are represented as mean ± S.D. for 3 experiments using different well in a single passage of Caco-2 cells.

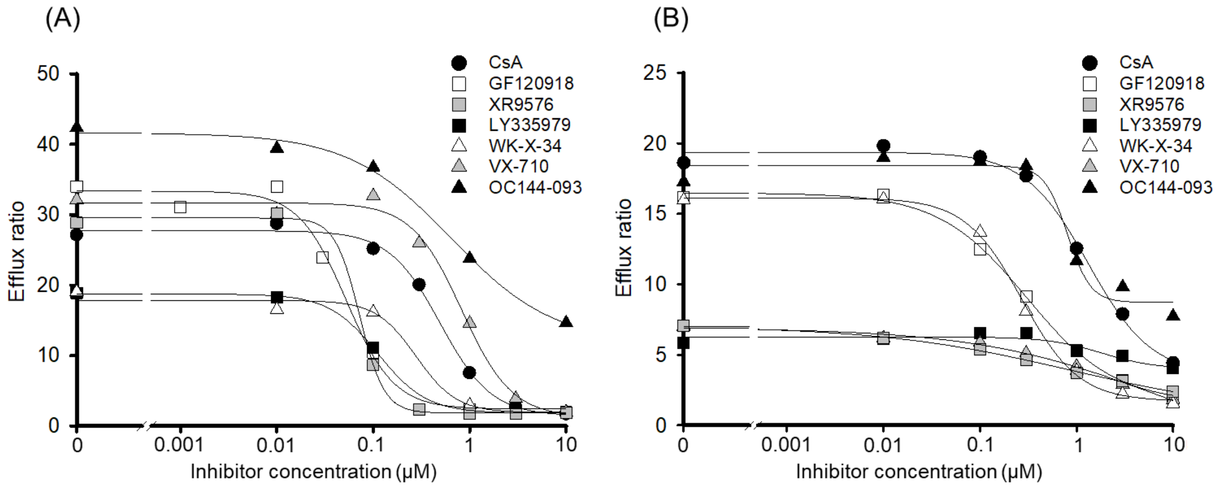

Figure S3. Efflux ratio (*ER*) of paclitaxel and mitoxantrone in Caco-2 cells with P-gp inhibitors. The *ER* values were calculated using the mean values of $P_{app,AB}$ and $P_{app,BA}$ of paclitaxel and mitoxantrone in Caco-2 cells in the presence or absence of various concentrations of P-gp inhibitors.

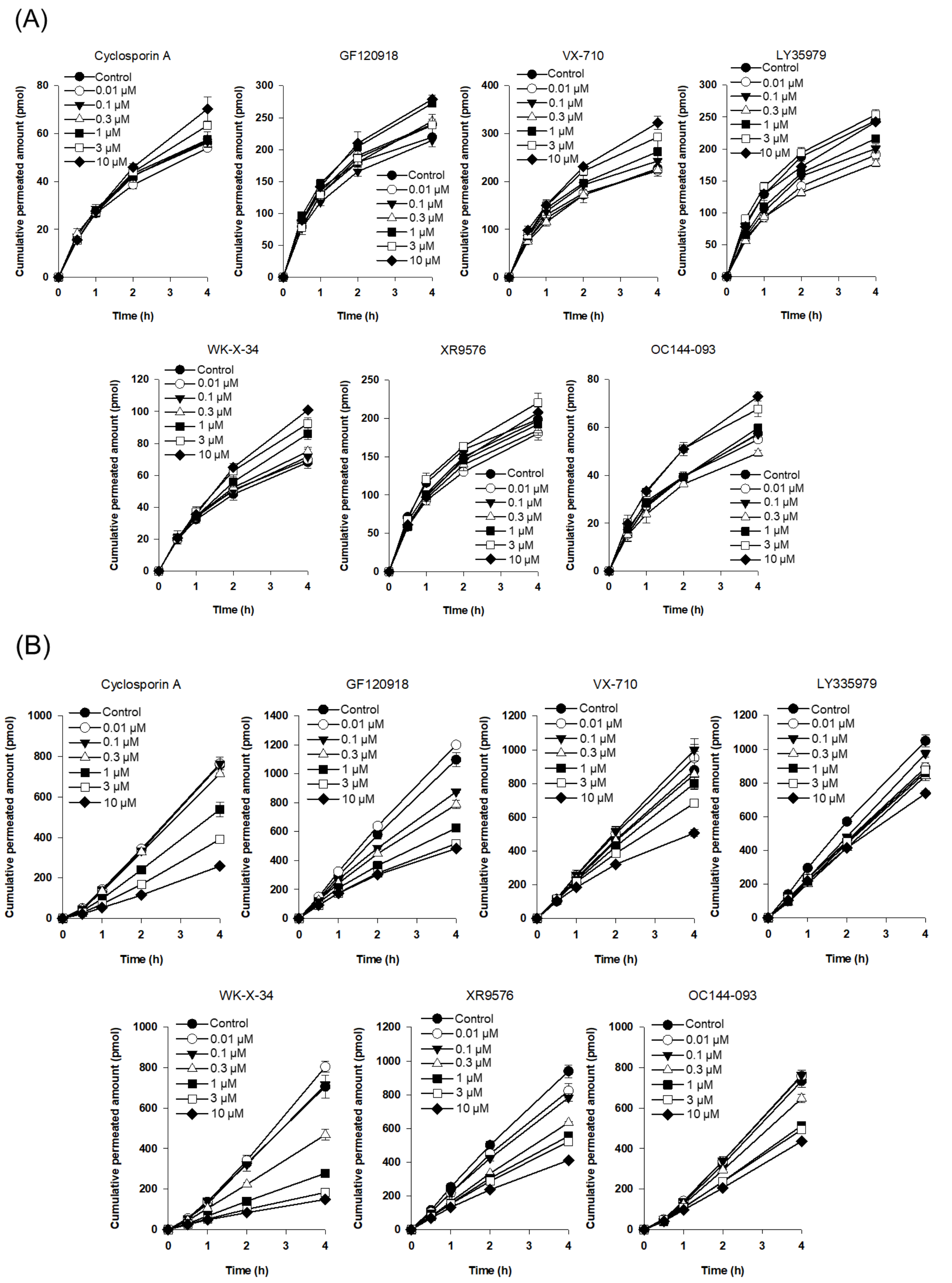

Figure S4. Bidirectional transport of mitoxantrone across Caco-2 cell monolayers. The apical-to-basal (A) and basal-to-apical (B) transport of mitoxantrone (5 μM) in the presence or absence of various concentrations of P-gp inhibitors. Data are represented as mean ± S.D. for 3 experiments using different well in a single passage of Caco-2 cells.
